# Improved Particle Swarm Optimization with a Collective Local Unimodal Search for Continuous Optimization Problems

**DOI:** 10.1155/2014/798129

**Published:** 2014-02-25

**Authors:** Martins Akugbe Arasomwan, Aderemi Oluyinka Adewumi

**Affiliations:** School of Mathematics, Statistics, and Computer Science, University of Kwazulu-Natal South Africa, Private Bag X54001, Durban 4000, South Africa

## Abstract

A new local search technique is proposed and used to improve the performance of particle swarm optimization algorithms by addressing the problem of premature convergence. In the proposed local search technique, a potential particle position in the solution search space is collectively constructed by a number of randomly selected particles in the swarm. The number of times the selection is made varies with the dimension of the optimization problem and each selected particle donates the value in the location of its randomly selected dimension from its personal best. After constructing the potential particle position, some local search is done around its neighbourhood in comparison with the current swarm global best position. It is then used to replace the global best particle position if it is found to be better; otherwise no replacement is made. Using some well-studied benchmark problems with low and high dimensions, numerical simulations were used to validate the performance of the improved algorithms. Comparisons were made with four different PSO variants, two of the variants implement different local search technique while the other two do not. Results show that the improved algorithms could obtain better quality solution while demonstrating better convergence velocity and precision, stability, robustness, and global-local search ability than the competing variants.

## 1. Introduction

Optimization comes to focus when there are needs to plan, take decisions, operate and control systems, design models, or seek optimal solutions to varieties of problems faced from day to day by different people. A number of these problems, which can be formulated as continuous optimization problems, are often approached with limited resources. Dealing with such problems, most especially when they are large scale and complex, has attracted the development of different nature-inspired optimization algorithms. These algorithms display problem-solving capabilities for researchers to solve complex and challenging optimization problems with many success stories. Swarm-based techniques are a family of nature-inspired algorithms and are population-based in nature; they are also known as evolutionary computation techniques. Particle swarm optimization (PSO) technique is a member of swarm-based techniques which is capable of producing low cost, fast, and robust solutions to several complex optimization problems. It is a stochastic, self-adaptive, and problem-independent optimization technique and was originally proposed in 1995 by Eberhart and Kennedy as simulation of a flock of bird or the sociological behavior of a group of people [1, 2]. From the time this concept was brought into optimization, it has been used extensively in many fields which include function optimization and many difficult real-world optimization problems [3–5].

PSO technique was initially implemented with few lines of codes using basic mathematical operations with no major adjustment needed to adapt it to new problems and it was almost independent of the initialization of the swarm [6]. It needs few parameters to operate with for successful and efficient behavior in order to obtain quality solutions. To implement this technique, a number of particles, which are characterized by positions and velocities, called swarm are required to be randomly distributed in a solution search space depending on the boundaries defined for the design variables of the problem being optimized. The number of design variables determines the dimensionality of the search space. If *d*-dimensional space is considered, the position and velocity of each particle are represented as the vectors *X*_*i*_ = (*x*_*i*1_,…, *x*_*id*_) and *V*_*i*_ = (*v*_*i*1_,…, *v*_*id*_), respectively. Every particle has a memory of its personal experience which is communicated to all reachable neighbours in the search space to guide the direction of movement of the swarm. Also, the quality of each particle (solution) is determined by the objective function of the problem being optimized and the particle with best quality is taken as the global solution towards which other particles will converge. The common practice is for the technique to maintain a single swarm of particles throughout its operation. This process of seeking optimal solution involves the adjustments of the position and velocity of each particle in each iteration using

(1) $$\begin{array}{c} V_{i}\left(t+1\right)=\omega V_{i}\left(t\right)+{\text{coeff}}_{1}\left(P_{i}-X_{i}\right)+{\text{coeff}}_{2}\left(P_{g}-X_{i}\right), \end{array}$$

(2) $$\begin{array}{c} X_{i}\left(t+1\right)=X\left(t\right)+V_{i}\left(t+1\right). \end{array}$$

In (1), *P*_*i*_ and *P*_*g*_ are vectors representing the *i*th particle personal best and swarm global best positions, respectively; coeff_1_ = *c*_1_ *r*_1_ and coeff_2_ = *c*_2_ *r*_2_; *c*_1_ and *c*_2_ are acceleration factors known as cognitive and social scaling parameters that determine the magnitude of the random forces in the direction of *P*_*i*_ and *P*_*g*_; *r*_1_ and *r*_2_ are random numbers between 0 and 1; *t* is iteration index. The symbol *ω* is the inertia weight parameter which was introduced into the original PSO in [7]. The purpose of its introduction was to help the PSO algorithm balance its global and local search activities.

There are possibilities of the positions and velocities of the particles in the swarm increasing in value beyond necessary when they are updated. As a measure, the positions are clamped in each dimension to the search range [*X*_min⁡_, *X*_max⁡_] of the design variables, where *X*_min⁡_ and *X*_max⁡_ represent the lower and upper bounds of a particle's position, respectively, while their velocities are controlled to be within a specified range [*V*_min⁡_, *V*_max⁡_], where *V*_min⁡_ and *V*_max⁡_ represent the lower and upper bounds of a particle's velocity, respectively. The idea of velocity clamping which was introduced by [1, 2, 8] and extensively experimented with in [9] has led to significant improvement as regards the performance of PSO. This is so because the particles could concentrate, taking reasonably sized steps to search through the search space rather than bouncing about excessively.

A major feature that characterizes an efficient optimization algorithm is the ability to strike a balance between local and global search. Global search involves the particles being able to advance from a solution to other parts of the search space and locate other promising candidates while local search means that the particle is capable of exploiting the neighbourhood of the present solution for other promising candidates. In PSO, as the rate of information sharing increases among the particles they migrate towards the same direction and region in the search space. If any of the particles could not locate any better global solution after some time, they will eventually converge about the existing one which may not be the global minimum due to lack of exploration power; this is known as premature convergence. This type of behaviour is more likely when the swarm of particles is overconcentrated. It could also occur when the optimization problem is of high dimension and/or nonconvex. One of the possible ways to prevent this premature convergence is to embed a local search technique into PSO algorithm to help improve the quality of each solution by searching its neighbourhood. After the improvement, better information is communicated among the particles thereby increasing the algorithm's ability to locate better global solution in course of optimization. Hill climbing, modified Hooke and Jeeves, gradient descent, golden ratio, Stochastic local search, adaptive local search, local interpolation, simulated annealing, and chaotic local search are different local search techniques that have been combined with PSO to improve its local search ability [10–18].

In this paper, a different local search technique was proposed to harness the global search ability of PSO and improve on its local search efforts. This technique is based on the collective efforts of randomly selected (with replacement) particles a number of times equal to the size of the problem dimension. When a particle is selected, it is made to contribute the value in the position of its randomly selected dimension from its personal best. The contributed values are then used to form a potential global best solution which is further refined. This concept could offer PSO the ability to enhance its performance in terms of convergence speed, local search ability, robustness, and increased solution accuracy. The local search technique was hybridized with two of the existing PSO variants, namely, random inertia weight PSO (RIW-PSO) and linear decreasing inertia weight PSO (LDIW-PSO), to form two new variants. Numerical simulations were performed to validate the efficiencies of each of them and some statistical analyses were performed to ascertain any statistically significant difference in performance between the proposed variants and the old ones. From the results obtained it was shown that the proposed variants are very efficient.

In the sections that follow, RIW-PSO and LDIW-PSO are briefly described in Section 2; the motivation and description of the proposed local search technique are presented in Section 3 while the improved PSO with local search technique is described in Section 4. Numerical simulations are performed in Section 5 and Section 6 concludes the paper.

## 2. The Particle Swarm Optimization Variants Used

Two PSO variants were used to validate the proposed improvement of the performance of PSO technique. The variants are LDIW-PSO and RIW-PSO. These were chosen because of the evidence available in the literature that they are less efficient in optimizing many continuous optimization problems [19–21]. These variants are succinctly described below.

### 2.1. PSO Based on Linear Decreasing Inertia Weight (LDIW-PSO)

This variant was proposed in [9] after the inertia weight parameter was introduced into the original PSO by [7]. It implements the linear decreasing inertia weight strategy represented in (3) which decreases from some high which facilitates exploration to a low value which on the other hand promotes exploitation. This greatly improved the performance of PSO. LDIW-PSO does global search at the beginning and converges quickly towards optimal positions but lacks exploitation power [9] and the ability required to jump out of the local minimum most especially when being in the multimodal landscape. Some improvements on LDIW-PSO exist in the literature [6, 9, 22]:

(3) $$\begin{array}{c} \omega_{\text{i}}=\left(\omega_{\text{start}}-\omega_{\text{stop}}\right)\left(\frac{{\text{MAX}}_{\text{itr}}-\text{i}}{{\text{MAX}}_{\text{itr}}}\right)+\omega_{\text{stop}}, \end{array}$$

where *ω*_start_ and *ω*_stop_ are the initial and final values of inertia weight, *i* is the current iteration number, MAX_itr_ is the maximum iteration number, and *ω*_i_ ∈ [0,1] is the inertia weight value in the *i*th iteration. Apart from the problem of premature convergence, this variant was found inefficient in tracking a nonlinear dynamic system because of the difficulty in predicting whether exploration (a larger inertia weight value) or exploitation (a smaller inertia weight) will be better at any given time in the search space of the nonlinear dynamic system [23].

### 2.2. PSO Based on Random Inertia Weight (RIW-PSO)

Due to the improved performance of PSO when the constant inertia weight was introduced into it [7], a new era of research was indirectly initiated and this has attracted the attentions of many researchers in the field. The inefficiency of linear decreasing inertia weight, which linearly decreases from 0.9 to 0.4, in tracking a nonlinear dynamic system prompted the introduction of RIW which randomly varies within the same range of values. Random adjustment is one of the strategies that have been proposed to determine the inertia weight value to further improve on the performance of PSO. This strategy is nonfeedback in nature and the inertia weight takes different value randomly at each iteration, from a specified interval. In line with this, random inertia weight strategy represented in (4) was introduced into PSO by [23] to enable the algorithm track and optimize dynamic systems. In the equation, rand() is a uniform random number in the interval [0,1] which make the formula generate a number randomly varying between 0.5 and 1.0, with a mean value of 0.75. When *c*_1_ and *c*_2_ are set to 1.494, the algorithm seems to demonstrate better optimizing efficiency. The motivation behind the selection of these values was Clerc's constriction factor [23]:

(4) $$\begin{array}{c} \omega =0.5+\frac{\text{rand}\left(\right)}{2}. \end{array}$$

Not much is recorded in the literature regarding the implementation of this variant of PSO. Some of the few implementations found in the literature are recorded in [6, 20–22].

## 3. Proposed Local Search Technique

The basic principle underlying the optimizing strategy of PSO technique is that each particle in the swarm communicates their discoveries to their neighbours and the particle with the best discovery attracts others. While this strategy looks very promising, there is the risk of the particles being susceptible to premature convergence, especially when the problem to be optimized is multimodal and high in dimensionality. The reason is that the more the particles share their discoveries among themselves, the higher their identical behaviour is until they converge to the same area in the solution search space. If none of the particle could discover better global best, after some time all the particles will converge about the existing global best which may not be the global minimizer.

One of the motivations for this local search technique is the challenge of premature convergence associated with PSO technique which affects its reliability and efficiency. Another motivation is the decision-making strategy used by the swarm in searching for optimal solution to optimization problems. The decision is dictated by a single particle in the swarm; that is, other particles follow the best particle among them to search for better solution. Involving more than one particle in the decision making could lead to a promising region in the search space where optimal solution could be obtained.

The description of the local search technique is as follows: after all the particles have obtained their various personal best positions, each particle has an equal chance of being selected to contribute its idea towards how a potential location in the search space where better global best could be obtained. As a result, a number of particles equal to the dimension of the problem being optimized are randomly selected (with replacement). Each selected particle contributes an idea by donating the value in the location of its randomly selected dimension from its personal best. All the ideas contributed by the selected particles are collectively used (hybridized) to construct a potential solution in the solution search space. After constructing the potential solution, some searches are locally done around its neighbourhood with the hope of locating a better solution in comparison with the current global solution. If a better solution is found, it is then used to replace the current global solution; otherwise no replacement is made.

In this local search, the potential new position is denoted by $\vec{y}$ and is sampled from the neighbourhood of the collectively constructed potential global solution represented as $\vec{P}$ by

(5) $$\begin{array}{c} \vec{y}⟵\vec{P}+\vec{a}, \end{array}$$

where $\vec{a}\sim U\left[-\vec{r},\vec{r}\right]$ is a random vector picked uniformly from the range $\left[-\vec{r},\vec{r}\right]$ and $\vec{r}$ is the search radius which is initially set to max*R* (maximum radius for local search). The local search technique moves from position $\vec{P}$ to position $\vec{y}$ when there is improvement to the fitness. If there is no improvement on the fitness of $\vec{P}$ by $\vec{y}$, the search radius is linearly decreased by multiplying it with a factor *q* using

(6) $$\begin{array}{c} \vec{r}⟵q\times \vec{r},\\ q⟵\left(\max R-\min R\right)\times \frac{t}{\max T}+\min R, \end{array}$$

where max*T* is the maximum number of times the neighbourhood of $\vec{P}$ is to be sampled, *t* is the current time the neighbourhood is being sampled, and min*R* is the minimum radius for the local search.

This proposed local search technique has been named collective local unimodal search (CLUS) technique. It has some trace of similarity in operation with local unimodal sampling (LUS) technique [24]. But they are quite different in the sense that, while LUS randomly picks a potential solution from the entire population, CLUS constructs a potential solution using the collective efforts of a randomly selected number of particles from the swarm. Also, CLUS uses a linear method to decrease the search radius (step size) in the neighbourhood of the potential solution which is different from the method applied by LUS during optimization. The CLUS technique is presented in Algorithm 1. In the technique, *g*Fit and $\vec{g\text{Pos}}$ represent the current global fitness value and its corresponding position in the search space.

## 4. Improved PSO with Collective Unimodal Local Search (PSO_**CLUS**_)

The RIW-PSO increases convergence in early iterations and does more of global search activities but soon gets stuck in local optima because of lack of local search ability. Also, LDIW-PSO does global search at earlier part of its iteration but lacks enough momentum to do local search as it gets towards its terminal point of execution. The aim of this paper is to make a general improvement on the performance of PSO which can be applied to any of its variants. To achieve this, the two PSO variants were hybridized with the proposed collective local unimodal search (CLUS) technique which takes advantage of their global search abilities to do some neighbourhood search for better results. The improved PSO algorithm is presented in Algorithm 2.

## 5. Numerical Simulations

In this section, the improved algorithm (PSO_CLUS_) was implemented using the inertia weight strategy of RIW-PSO and the variant was labeled *R*-PSO_CLUS_. It was also implemented using the inertia weight strategy of LDIW-PSO and the variant was labeled *L*-PSO_CLUS_. The performances of *R*-PSO_CLUS_ and *L*-PSO_CLUS_ were experimentally tested against those of RIW-PSO and LDIW-PSO, respectively. The maximum number of iterations allowed was 1000 for problems with dimensions less than or equal to 10, 2000 for 20-dimensional problems, and 3000 for 30-dimensional problems. A swarm size of 20 was used in all the experiments and twenty-five independent runs were conducted to collect data for analysis. The termination criteria for all the algorithms were set to be as maximum number of iterations relative to the problems' dimensions. A run, in which an algorithm is able to satisfy the set success criteria (see Table 1) before or at the maximum iteration, is considered to be successful. To further prove the efficiency of the proposed local search technique, the proposed PSO variants were also compared with some existing PSO variants hybridized with different local search techniques. They are PSO with golden ratio local search [15] and PSO with local interpolation search [18]. A total of 6 different experiments were conducted.

- *R*-PSO_CLUS_ was compared with PSO with golden ratio local search (GLSPSO);
- *R*-PSO_CLUS_ was compared with PSO with local interpolation search (PSOlis);
- *R*-PSO_CLUS_ was compared with RIW-PSO;
- *L*-PSO_CLUS_ was compared with PSO with golden ratio local search (GLSPSO);
- *L*-PSO_CLUS_ was compared with PSO with local interpolation search (PSOlis);
- *L*-PSO_CLUS_ was compared with LDIW-PSO.

The application software was developed in Microsoft Visual C# programming language.

### 5.1. Test Problems

A total of 21 problems were used in the experiments. These problems have different degrees of complexity and multimodality which represents diverse landscapes enough to cover many of the problems which can arise in global optimization problems. Shown in Table 2 are the problems dimensions, optimal fitness values, and success thresholds. Presented in Table 3 are the definitions, characteristics (US: unimodal separable, UN: unimodal nonseparable, MS: multimodal separable, and MN: multimodal nonseparable), and search ranges of the problems. More details on the benchmark problems can be found in [22, 25–27].

### 5.2. Parameter Setting

The additional parameters that were set in the experiment are inertia weight threshold for LDIW-PSO (*ω*_min⁡_ and *ω*_max⁡_), acceleration coefficients (*c*_1_ and *c*_2_), velocity thresholds (*V*_min⁡_ and *V*_max⁡_), minimum radius (min*R*), and maximum radius (max*R*) for local search as well as the maximum number of neighbourhood sampling (max*T*) during the local search. The respective settings of these parameters are shown in Table 1. The parameters *r*_1_ and *r*_2_ were randomly generated using the uniform random number generator. The values of *ω*_min⁡_ and *ω*_max⁡_ were chosen for LDIW-PSO based on the experiments conducted in [9]; values for *c*_1_ and *c*_2_ were chosen for RIW-PSO based on the recommendation in [23] and it was also used for LDIW-PSO because it was discovered in course of the experiments in this paper that these values make LDIW-PSO perform better than the commonly used value of 2.0. The settings for *V*_min⁡_ and *V*_max⁡_ were done based on the outcome of experimental studies in [8].

### 5.3. Performance Measurement

The efficiency of the algorithms was tested against the set of benchmark problems given in Table 2 and numerical results obtained were analyzed using the criteria that are listed below. All the results are presented in Tables 4 to 20.

- Best fitness solution: the best of the fitness solution among the solutions obtained during the runs.
- Mean best fitness solution: this is a measure of the precision (quality) of the result that the algorithm can get within given iterations in all the 25 runs.
- Standard deviation (Std. Dev.) of mean best fitness solution over 25 runs: this measures the algorithm's stability and robustness.
- Average number of iterations an algorithm was able to reach the success threshold.
- Success rate (SR) = (Number of successful runs/Total number of runs) × 100: this is the rate at which the success threshold is met during the independent number of runs and is a reflection of the global search ability and robustness of the algorithm.

Statistical analysis using the Wilcoxon signed rank nonparametric test with 0.05 level of significance [28, 29] was also performed using the numerical results obtained by the algorithms, while box plots were used to analyze their variability in obtaining fitness values in all the runs.

### 5.4. Results and Discussions

Results obtained from all the experiments are discussed in this subsection to show the overall performance of the various algorithms. Presented in Tables 4, 5, 6, 7, 8, and 9 are the numerical results obtained and used to compare *R*-PSO_CLUS_ and *L*-PSO_CLUS_ with GLSPSO. *R*-PSO_CLUS_ and *L*-PSO_CLUS_ were also compared with PSOlis using the results presented in Table 10. The results in Tables 11–18 were obtained for the scaled and nonscaled test problems listed in Table 3; the results were used to validate RIW-PSO, *R*-PSO_CLUS_, LDIW-PSO, and *L*-PSO_CLUS_. In each of the tables, for ease of observation, bold values represent the better results and “–” means that the algorithm could not satisfy the success threshold in any of the runs. The Wilcoxon sign rank nonparametric test, which is used as an alternative to the paired *t*-test when the results cannot be assumed to be normally distributed, was applied to test the statistical significance differences between RIW-PSO and *R*-PSO_CLUS_ as well as LDIW-PSO and *L*-PSO_CLUS_.

#### 5.4.1. Comparison of *R*-PSO_CLUS_ and Golden Ratio Local Search Based PSO (GLSPSO)

The results in Tables 4–6 show the performance and abilities of *R*-PSO_CLUS_ and GLSPSO optimizing the test problems over three different problem dimensions. The results of GLSPSO were obtained from [15]. A large problem space was used for all the problems to verify the superiority between the two different local search techniques hybridized with the PSO variants. From the results it is evident that *R*-PSO_CLUS_ is superior to GLSPSO. Apart from *Ackley* problem (across the three dimensions) and *Rosenbrock* (in dimension 100), *R*-PSO_CLUS_ outperformed GLSPSO. It was able to obtain optimal minimum for some of the problems, demonstrating better exploitation ability, convergence precision, and solution quality.

#### 5.4.2. Comparison between *L*-PSO_CLUS_ and GLSPSO

To further demonstrate the efficiency of the proposed local search technique, *L*-PSO_CLUS_ was also implemented and results were compared with the results of GLSPSO obtained from [15]. Three different types of dimensions were also used for the problems. As can be observed in Tables 7–9, across the three dimensions, GLSPSO was only able to perform better than *L*-PSO_CLUS_ in *Ackley* problem. Apart from *Griewank* problem (dimension 10), GLSPSO was outperformed by *L*-PSO_CLUS_ in the remaining four problems. *L*-PSO_CLUS_ was able to obtain global optimum for *Griewank* and *Sphere* problems across the three dimensions, but, for *Rastrigin*, it was able to get global minimum for dimension 10. Again, the proposed local search technique demonstrates better exploitation ability than GLSPSO.

#### 5.4.3. Comparison of *R*-PSO_CLUS_ and *L*-PSO_CLUS_ with PSOlis

Presented in Table 10 is the result obtained by *L*-PSO_CLUS_ in comparison with result for PSOlis from [18]. Again, the outstanding performance of *L*-PSO_CLUS_ over its competitor is evidence that the proposed local search technique is very efficient and capable of complementing the global search ability of PSO to obtain quality results by making it overcome premature convergence.

#### 5.4.4. Comparison between RIW-PSO and *R*-PSO_CLUS_

**Figure 1:**
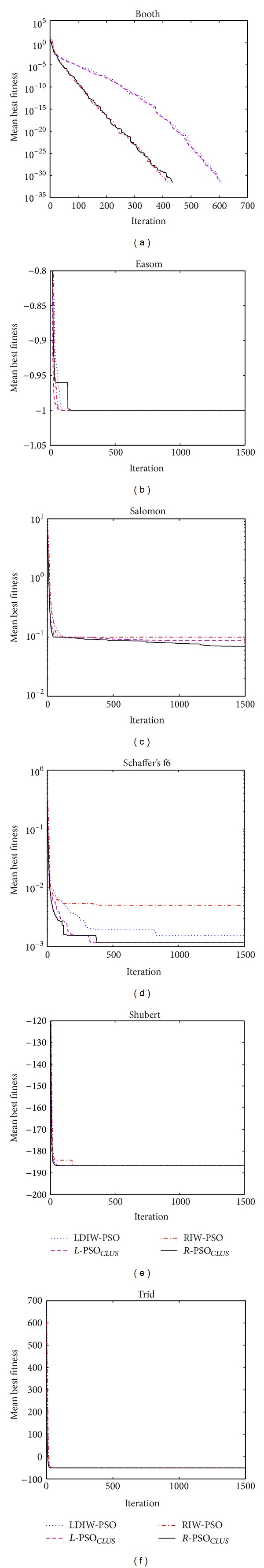
Convergence graphs for 6 of the nonscaled benchmark problems.

**Figure 2:**
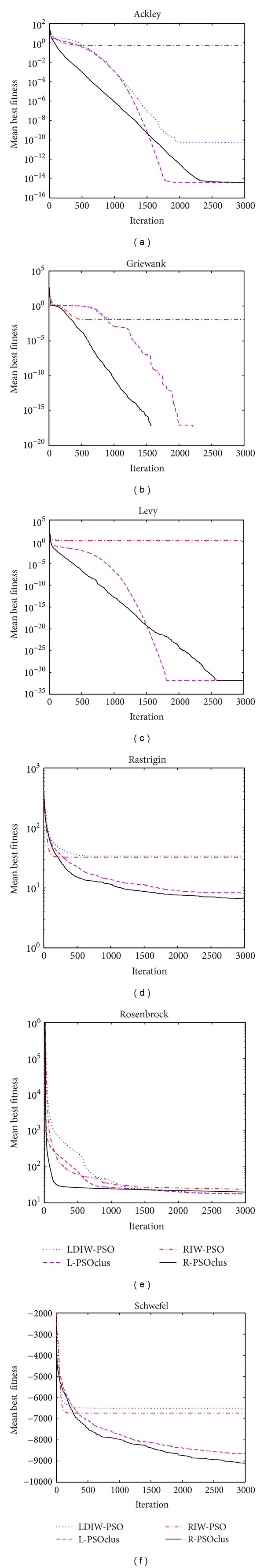
Convergence graphs for 6 of the scaled benchmark problems with dimension of 30.

The results presented in Table 11 are for the nonscaled test problems as optimized by the two algorithms while those in Tables 12–14 are for the scaled problems with 10, 20, and 30 dimensions, respectively. In Table 19 are the results obtained using the Wilcoxon sign rank nonparametric test.

(*1)  Results for the Nonscaled Problems*. For the 7 nonscaled problems, Table 11 shows that there are no performance differences between the two algorithms in optimizing *Booth*, *Easom*, *Shubert,* and *Trid* problems. For *Michalewicz*, *Schaffer's f6*, and *Salomon*, *R*-PSO_CLUS_ obtained more quality solutions and demonstrated better global search ability than RIW-PSO. The convergence curves in Figures 1(c) and 1(d) show that *R*-PSO_CLUS_ has faster and better convergence. However, the *P* value (0.190) obtained from the Wilcoxon sign test shown in Table 19 revealed that there is no statistical difference in the performance between the two algorithms for the nonscaled problems. Also, the two algorithms have equal median fitness.

(*2)  Results for 10-Dimensional Problems*. For the scaled problems with 10 dimensions, Table 12 clearly reveals great differences in performance between RIW-PSO and *R*-PSO_CLUS_. The two algorithms successfully optimized *Rastrigin*, *Rosenbrock*, *Rotated Ellipsoid*, *Schwefel 2.22*, *Sphere,* and *Sum Squares* problems with equal success rate of 100%, but *R*-PSO_CLUS_ obtained significantly better mean fitness and standard deviation with fewer number of iterations. *R*-PSO_CLUS_ was able to obtain the minimum optima for both *Rastrigin* and *Step* problems. For the other problems, *R*-PSO_CLUS_ clearly outperformed RIW-PSO in solution quality, convergence precision, global search ability, and robustness, though none of them could meet the success threshold in optimizing the *Noisy Quartic* problem. The *P* value (0.001) obtained from the Wilcoxon sign test presented in Table 19 indicates that there is statistically significant difference in performance between the two algorithms with a large effect size of *r* = 0.6 in favour of *R*-PSO_CLUS_. The median fitness is also an evidence of this.

(*3)  Results for 20-Dimensional Problems*. The same set of experiments was performed using the same scaled problems but with their dimensions increased to 20, which also increased their complexities except *Griewank*. The numerical results in Table 13 also show that there are great differences in performance between RIW-PSO and *R*-PSO_CLUS_. The two algorithms had equal success rate of 100% in optimizing *Rosenbrock*, *Schwefel 2.22*, *Sphere,* and *Sum Squares* problems with *R*-PSO_CLUS_ obtaining significantly better mean fitness (except *Rosenbrock*), standard deviation, and fewer number of iterations. Out of the remaining 10 problems *R*-PSO_CLUS_ outperformed RIW-PSO in 9 of them with better solution quality, convergence precision, global search ability, and robustness; it also had success rate of 100% in 6 of the problems compared with RIW-PSO and was able to obtain global minimum for *Griewank* and *Step* problems. The algorithms could not meet the success criteria in optimizing the *Dixon-Price*, *Noisy Quartic,* and *Schwefel* problems, but *R*-PSO_CLUS_ still performed better than RIW-PSO. The *P* value (0.023) from Wilcoxon sign test as shown in Table 19 also indicates that there is statistically significant difference in performance between the two algorithms with a medium effect size of *r* = 0.43 in favour of *R*-PSO_CLUS_. The median fitness value of *R*-PSO_CLUS_ is smaller than that of RIW-PSO.

(*4)  Results for 30-Dimensional Problems*.  Table 14 represents the experimental results obtained by the two algorithms using the same scaled problems but with their dimensions scaled to 30, which further increased their complexities except *Griewank*. The results further show the great differences in performance between RIW-PSO and *R*-PSO_CLUS_. Out of the 14 problems *R*-PSO_CLUS_ had 100% success rate in 7 of them (4 multimodal and 3 unimodal) while RIW-PSO could only have in 3 of them (all unimodal). The two algorithms had equal success rate of 100% in optimizing *Schwefel 2.22*, *Sphere,* and *Sum Squares* problems with *R*-PSO_CLUS_ obtaining significantly better mean fitness standard deviation and fewer number of iterations. Optimizing *Dixon-Price*, *Noisy Quartic*, *Rotated Ellipsoid,* and *Schwefel* problems, none of the algorithms could meet the success criteria, yet *R*-PSO_CLUS_ still obtained better results than RIW-PSO. In all the 14 problems except *Rotated Ellipsoid*, *R*-PSO_CLUS_ outperformed RIW-PSO and was able to obtain global minimum for *Griewank* and *Step* problems. The *P* value (0.009) from Wilcoxon sign test in Table 19 is a confirmatory evidence that there is statistically significant difference in performance between RIW-PSO and *R*-PSO_CLUS_ with a large effect size of *r* = 0.49 in favour of *R*-PSO_CLUS_. The median fitness value of *R*-PSO_CLUS_ is also smaller than that of RIW-PSO. The convergence graphs of six 30-dimensional test problems shown in Figure 2 demonstrate the speed and ability of convergence of the two algorithms. From the graphs it is clear that *R*-PSO_CLUS_ demonstrates better convergence and global search ability than RIW-PSO. Besides it also possesses better ability to get out of local optima.

#### 5.4.5. Comparison between LDIW-PSO and *L*-PSO_CLUS_

Presented in Table 15 are the results for the nonscaled test problems as optimized by the two algorithms while those in Tables 16–18 are for the scaled problems with 10, 20, and 30 dimensions, respectively. The statistical analysis done by applying Wilcoxon sign rank nonparametric test is presented in Table 20.

(*1)  Results for the Nonscaled Problems*. Results in Table 15 show that there are no clear performance differences between LDIW-PSO and *L*-PSO_CLUS_ in optimizing *Booth*, *Easom*, *Shubert,* and *Trid* problems; however, there are some not too significant differences in their average number of iterations to reach the success thresholds and standard deviation; in *Shubert*, LDIW-PSO obtained 100% success but *L*-PSO_CLUS_ could not. Figures 1(a), 1(b), 1(e), and 1(f) show their convergence behaviour. Optimizing *Michalewicz*, *Schaffer's f6*, and *Salomon*, *L*-PSO_CLUS_ obtained better quality solutions and has better search ability than LDIW-PSO. Also, the convergence graphs in Figures 1(c) and 1(d) show that  *L*-PSO_CLUS_ have faster and better convergence in *Schaffer's f6* and *Salomon*. The curves show that the two algorithms were trapped in local optima as shown by the flat parts of their curves and were able to escape from some of them. The *P* value (0.190) in Table 20, obtained from the Wilcoxon sign test, indicates that there is no statistically significant difference between the two algorithms in performance.

(*2)  Results for 10-Dimensional Problems*. Optimizing the 10-dimensional scaled problems, *L*-PSO_CLUS_ had 100% success in 10 of the 14 problems (4 multimodal and 6 unimodal) while LDIW-PSO had 100% success in 6 problems (1 multimodal and 5 unimodal) as shown in Table 16. It is only *L*-PSO_CLUS_ that could successfully obtain the minimum optima for both *Rastrigin* and *Step* problems but none could reach the success threshold for *Dixon*-*Price* and *Noisy Quartic*. In all the problems except *Dixon*-*Price* (where they have approximately equal performance) and *Sum Squares*, *L*-PSO_CLUS_ clearly outperformed LDIW-PSO in obtaining better solution quality, convergence precision, global search ability, and robustness as well as fewer number of iterations. To confirm this, Wilcoxon sign test was performed on the mean best fitness over all the problems and results are presented in Table 20; the *P* value (0.003) obtained indicates that there is statistically significant difference in performance between the two algorithms with a large effect size of *r* = 0.565 in favour of *L*-PSO_CLUS_ which also has a lower median value for the mean best fitness.

(*3)  Results for 20-Dimensional Problems*. The numerical results in Table 17 also show that there are great differences in performance between LDIW-PSO and *L*-PSO_CLUS_ performing the same set of experiments but with the problems dimensions increased to 20. The two algorithms had equal success rate of 100% in optimizing *Ackley*, *Rosenbrock*, *Schwefel 2.22*, *Sphere,* and *Sum Squares* problems with *L*-PSO_CLUS_ obtaining significantly better mean fitness (except *Rosenbrock*), standard deviation, and fewer number of iterations. *L*-PSO_CLUS_ outperformed LDIW-PSO in 7 (5 multimodal and 2 unimodal) of the rest 9 problems and obtained better solution, convergence precision, global search ability, and robustness; it was also able to obtain global minimum for *Step* problem. The algorithms could not reach the success thresholds for *Dixon-Price*, *Noisy Quartic,* and *Schwefel* problems. The nonparametric test that was performed using Wilcoxon sign test, with results shown in Table 20, also confirms statistically significant difference in performance between the two algorithms with *P* value (0.011) and a large effect size of *r* = 0.482 in the direction of *L*-PSO_CLUS_. The median fitness value of *L*-PSO_CLUS_ is also smaller than that of LDIW-PSO.

(*4) Results for 30-Dimensional Problems*. Scaling the dimensions of test problems to 30 to further increase their complexities, except *Griewank *which decreases in complexity with increased dimension, did not affect the better performance of *L*-PSO_CLUS_ over LDIW-PSO. Presented in Table 18 are the experimental results obtained by the two algorithms optimizing the same scaled problems. The results indicate that there are great differences between LDIW-PSO and *L*-PSO_CLUS_ in performance. Out of the 14 problems *L*-PSO_CLUS_ had 100% success rate in 6 of them (3 multimodal and 3 unimodal) while LDIW-PSO could only have in 3 (1 multimodal and 2 unimodal). They had equal success rate of 100% in optimizing *Ackley*, *Sphere,* and *Sum Squares* problems and 92% in *Rosenbrock* with *L*-PSO_CLUS_ obtaining significantly better results. Optimizing *Dixon-Price*, *Noisy Quartic,* and *Schwefel* problems, none of the algorithms could reach the success threshold, yet *L*-PSO_CLUS_ still obtained better results than LDIW-PSO, except in *Dixon-Price* where they had approximately the same performance. LDIW-PSO was not able to reach success threshold for *Noncontinuous Rastrigin* and *Rotated Ellipsoid* problems unlike *L*-PSO_CLUS_. In all the 14 problems *L*-PSO_CLUS_ conceded in none to LDIW-PSO and it was able to obtain global minimum for *Griewank* and *Step* problems. The *P* value (0.001) in Table 20 further confirms that there is statistically significant difference between LDIW-PSO and *L*-PSO_CLUS_ with a large effect size of *r* = 0.601 in the direction of *L*-PSO_CLUS_. The median value for the mean fitness of *L*-PSO_CLUS_ is also smaller than that of RIW-PSO.  Figure 1 shows the convergence graphs of the two algorithms. From the graphs it is clear that *L*-PSO_CLUS_ demonstrates better convergence speed, better ability to escape premature convergence, and global search ability than LDIW-PSO.

#### 5.4.6. Box Plots Analysis

**Figure 3:**
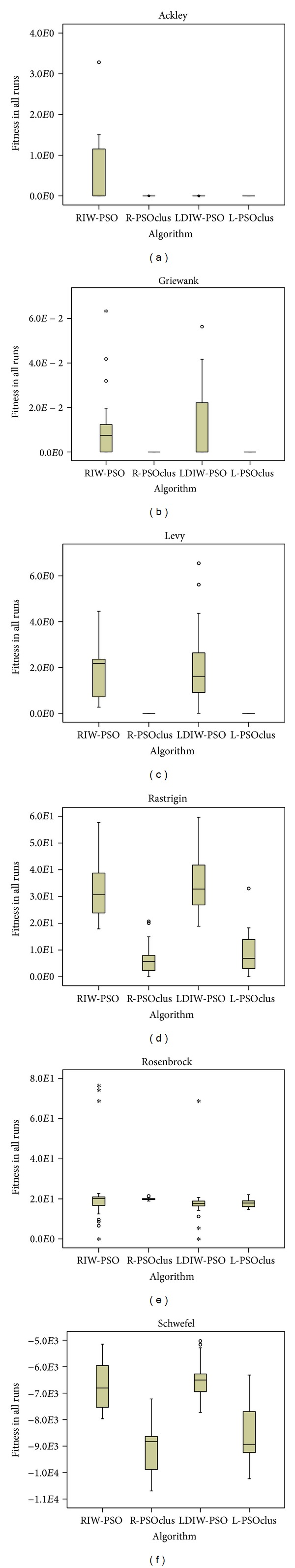
Box plots for 6 of the scaled test problems.

Other than using statistical test to observe the performance of RIW-PSO, *R*-PSO_CLUS_, LDIW-PSO, and *L*-PSO_CLUS_, box plots analysis was also performed for 6 of the scaled test problems with 30 dimensions; the results are presented in Figures 3(a)–3(f). Box plots give a direct visual comparison of both location and the dispersion of data. The four algorithms are plotted together to optimize space. In each of the plot, RIW-PSO is compared with *R*-PSO_CLUS_ while LDIW-PSO is compared with *L*-PSO_CLUS_. The plots strengthen and justify the better performance of PSO when used with the proposed local search technique.

## 6. Conclusion

A new local search technique has been proposed in this paper with the goal of addressing the problem of premature convergence associated with particle swarm optimization algorithms. The proposed local search was used to efficiently improve the performance of two existing PSO variants, RIW-PSO and LDIW-PSO. These variants have been known to be less efficient optimizing continuous optimization problems. In this paper they were hybridized with the local search to form two other variants *R*-PSO_CLUS_ and *L*-PSO_CLUS_. Some well-studied benchmark problems with low and high dimensions were used to extensively validate the performance of these new variants and comparisons were made with RIW-PSO and LDIW-PSO. They were also compared with two other PSO variants in the literature, which are hybridized with different local search techniques. The experimental results obtained show that the proposed variants successfully obtain better results with high quality while demonstrating better convergence velocity and precision, stability, robustness, and global-local search ability than the competing variants. This therefore shows that the local search technique proposed can help PSO algorithms execute effective exploitation in the search space to obtain high quality results for complex continuous optimization problems. This local search technique can be used with any population-based optimization algorithms to obtain quality solutions to simple and complex optimization problem.

Further study is needed on the parameter tuning of the proposed local search technique. Empirical investigation of the behaviour of the technique in optimizing problems with noise needs further study. The scalability of the algorithms for problems with higher dimension greater than 100 is essential. Finally, the proposed algorithm can be applied to real-world optimization problems.

## Figures and Tables

**Algorithm 1:**
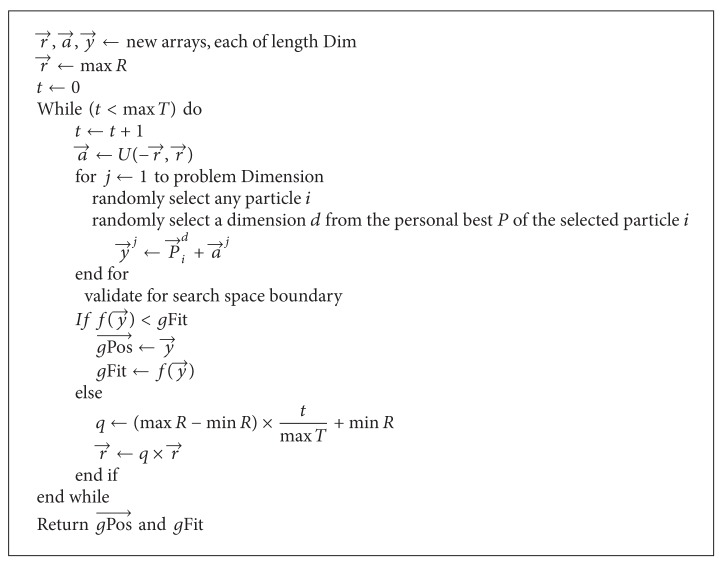
Collective local unimodal search.

**Algorithm 2:**
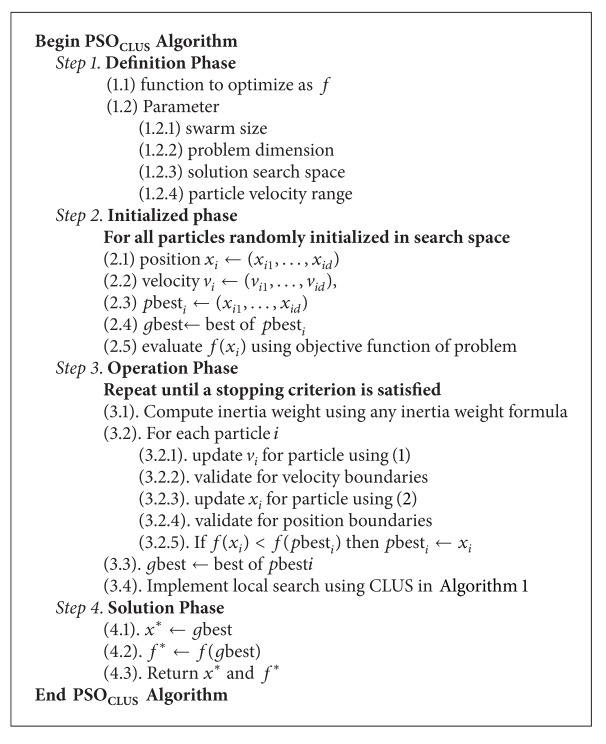
Algorithm for PSO_CLUS_.

**Table 1:** Parameter settings for experiment.

Parameter	*ω*_min⁡_	*ω*_max⁡_	*c*_1_ = *c*_2_	*V*_min⁡_	*V*_max⁡_	min*R *	max*R *	max*T *
Value	0.9	0.4	1.494	0.05 ∗ *X*_min⁡_	0.05 ∗ *X*_max⁡_	0.01	2.0	100

**Table 2:** Benchmark problems.

Number	Problem	Dimensions	Optimal value	Success threshold
1	Ackley	10, 20, 30	0	10^−5^
2	Booth	2	0	10^−5^
3	Easom	2	−1	−1
4	Griewank	10, 20, 30	0	10^−5^
5	Dixon-Price	10, 20, 30	0	10^−5^
6	Levy	10, 20, 30	0	10^−5^
7	Michalewicz	5	−4.687	−4.687
8	Noisy Quartic	10, 20, 30	0	10^−5^
9	Noncontinous Rastrigin	10, 20, 30	0	20
10	Rastrigin	10, 20, 30	0	20
11	Rosenbrock	10, 20, 30	0	20
12	Rotated Ellipsoid	10, 20, 30	0	10^−5^
13	Salomon	5	0	10^−5^
14	Schaffer's f6	2	0	10^−5^
15	Schwefel	10, 20, 30		
16	Schwefel P2.22	10, 20, 30	0	10^−5^
17	Shubert	2	−186.7309	−186.7309
18	Sphere	10, 20, 30	0	10^−5^
19	Step	10, 20, 30	0	10^−5^
20	Sum Squares	10, 20, 30	0	10^−5^
21	Trid	6	−50	−50

**Table 3:** Benchmark problems.

Number	Problem	Formulation	Feature	Search range
1	Ackley	$f\left(\vec{x}\right)=-20\exp\left(-0.2\sqrt{\frac{1}{n}\overset{d}{\underset{i=1}{\sum }}x_{i}^{2}}\right)-\exp\left(\frac{1}{n}\overset{d}{\underset{i=1}{\sum }}\cos\left(2\pi x_{i}\right)\right)+20+e$	MN	±32
2	Booth	$f\left(\vec{x}\right)={\left(x_{1}+2x_{2}-7\right)}^{2}+{\left(2x_{1}+x_{2}-5\right)}^{2}$	MN	±10
3	Easom	$f\left(\vec{x}\right)=-\cos\left(x_{1}\right)\cos\left(x_{2}\right)\exp\left(-{\left(x_{1}-\pi \right)}^{2}-{\left(x_{2}-\pi \right)}^{2}\right)$	UN	±100
4	Griewank	$f\left(\vec{x}\right)=\frac{1}{4000}\left(\overset{d}{\underset{i=1}{\sum }}x_{i}^{2}\right)-\left(\prod_{i=1}^{d}\cos\left(\frac{x_{i}}{\sqrt{i}}\right)\right)+1$	MN	±600
5	Dixon-Price	$f\left(\vec{x}\right)={\left(x_{1}-1\right)}^{2}+\overset{d}{\underset{i=2}{\sum }}i{\left(2x_{i}^{2}-x_{i-1}\right)}^{2}$	UN	±10
6	Levy	$f\left(\vec{x}\right)=\sin^{2}\left(\pi y_{1}\right)+\overset{d-1}{\underset{i=1}{\sum }}{\left(y_{i}-1\right)}^{2}\left(1+10\;\sin^{2}\left(\pi y_{i}+1\right)\right)+{\left(y_{d}-1\right)}^{2}\left(1+\sin^{2}\left(2\pi x_{d}\right)\right),$ where $\;y_{i}=1+\frac{x_{i}-1}{4},\;\text{and}\;\;i=1,2,\ldots ,d$	MN	±10
7	Michalewicz	$f\left(\vec{x}\right)=-\overset{d}{\underset{i=1}{\sum }}\sin\left(x_{i}\right){\left[\sin\left(\frac{ix_{i}^{2}}{\pi }\right)\right]}^{2m},\;\text{where}\;\;m=10$	MS	[0, *π*]
8	Noisy Quartic	$f\left(\vec{x}\right)=\overset{d}{\underset{i=1}{\sum }}ix_{i}^{4}+\text{random}(0,1)$	US	±1.28
9	Noncontinous Rastrigin	$f\left(\vec{x}\right)=\overset{d}{\underset{i=1}{\sum }}\left(y_{i}^{2}-10\cos\left(2\pi y_{i}\right)+10\right)$ $y_{i}=\left\{\begin{array}{cc} x_{i} & \text{if }\left|x_{i}\right|<0.5\\ \frac{\text{round}(2x_{i})}{2} & \text{if }\left|x_{i}\right|\geq 0.5 \end{array}\right\}$	MS	±5.12
10	Rastrigin	$f\left(\vec{x}\right)=\overset{d}{\underset{i=1}{\sum }}\left(x_{i}^{2}-10\cos\left(2\pi x_{i}\right)+10\right)$	MS	±5.12
11	Rosenbrock	$f\left(\vec{x}\right)=\overset{d-1}{\underset{i=1}{\sum }}\left(100{\;\left(x_{i+1}-x_{i}^{2}\right)}^{2}\right)+{\left(x_{i}-1\right)}^{2}$	UN	±30
12	Rotated Ellipsoid	$f\left(\vec{x}\right)=\overset{d}{\underset{i=1}{\sum }}{\left(\overset{i}{\underset{j=1}{\sum }}x_{j}\right)}^{2}$	UN	±100
13	Salomon	$f\left(\vec{x}\right)=-\cos\left(2\pi \overset{d}{\underset{i=1}{\sum }}x_{i}^{2}\right)+0.1\sqrt{\overset{d}{\underset{i=1}{\sum }}x_{i}^{2}}+1$	MN	±100
14	Schaffer's f6	$f\left(\vec{x}\right)=\overset{d-1}{\underset{i=1}{\sum }}\left(0.5+\frac{\sin^{2}\left(\sqrt{x_{i+1}^{2}+x_{i}^{2}}\right)-0.5}{{\left(0.001\left(x_{i+1}^{2}+x_{i}^{2}\right)+1\right)}^{2}}\right)$	MN	±100
15	Schwefel	$f\left(\vec{x}\right)=\overset{n}{\underset{i=1}{\sum }}-x_{i}\sin\left(\sqrt{\left|x_{i}\right|}\right)$	MS	±500
16	Schwefel P2.22	$f\left(\vec{x}\right)=\overset{d}{\underset{i=1}{\sum }}\left|x_{i}\right|+\prod_{i=1}^{d}\left|x_{i}\right|$	UN	±10
17	Shubert	$f\left(\vec{x}\right)=\prod_{i=1}^{d}\left(\overset{5}{\underset{j=1}{\sum }}j\cos\left(\left(j+1\right)sx_{i}+j\right)\right)$	MN	±10
18	Sphere	$f\left(\vec{x}\right)=\overset{d}{\underset{i=1}{\sum }}x_{i}^{2}$	US	±100
19	Step	$f\left(\vec{x}\right)=\overset{d}{\underset{i=1}{\sum }}{\left(\left\lfloor x_{i}+0.5\right\rfloor \right)}^{2}$	US	±10
20	SumSquares		US	±10
21	Trid	$f\left(\vec{x}\right)=\overset{d}{\underset{i=1}{\sum }}{\left(x_{i}-1\right)}^{2}-\overset{d}{\underset{i=2}{\sum }}x_{i}x_{i-1}$	UN	±*d* ^2^

**Table 4:** Comparison between GLSPSO and *R*-PSO_CLUS_ for problems with dimension of 10.

Problem	Ackley		Griewank		Rastrigin		Rosenbrock		Sphere	
Algorithm	GLSPSO	*R*-PSO_CLUS_	GLSPSO	*R*-PSO_CLUS_	GLSPSO	*R*-PSO_CLUS_	GLSPSO	*R*-PSO_CLUS_	GLSPSO	*R*-PSO_CLUS_

Best fitness	0.0364	**0.0000**	4.2879*e* − 04	0.0000**e** + 00	8.8062	**0.0000**	2.6188	**0.0000**	4.7832*e* − 04	3.1461**e** − 43
Mean fitness	**0.3413**	17.1371	0.0041	**0.0016**	29.4936	**0.0000**	9.0025	**1.9971**	0.0142	**0.0000**
Worst fitness	**1.2653**	20.0888	**0.0419**	0.0791	50.4781	**0.0000**	18.9887	**3.1444**	0.0476	**0.0000**
Std. Dev.	**0.2762**	6.7543	**0.0061**	0.0111	10.4372	**0.0000**	0.034	**0.7262**	0.0123	**0.0000**

**Table 5:** Comparison between GLSPSO and *R*-PSO_CLUS_ for problems with dimension of 30.

Problem	Ackley		Griewank		Rastrigin		Rosenbrock		Sphere	
Algorithm	GLSPSO	R-PSO_CLUS_	GLSPSO	*R*-PSO_CLUS_	GLSPSO	*R*-PSO_CLUS_	GLSPSO	*R*-PSO_CLUS_	GLSPSO	*R*-PSO_CLUS_

Best fitness	**2.2784**	20.3075	0.0897	**0.0000**	109.5946	**13.9247**	175.8785	**22.7589**	1.9123	**0.0000**
Mean fitness	**2.8398**	20.4778	0.1257	**0.0000**	185.5221	**36.3715**	218.4976	**27.5147**	2.7449	**0.0000**
Worst fitness	**3.2952**	20.5792	0.2074	**0.0000**	229.6229	**72.6581**	259.2466	**76.7433**	3.9559	**0.0000**
Std. Dev.	0.2273	**0.0574**	0.0274	**0.0000**	24.9829	**16.4882**	21.8027	**9.9182**	0.4840	**0.0000**

**Table 6:** Comparison between GLSPSO and *R*-PSO_CLUS_ for problems with dimension of 100.

Problem	Ackley		Griewank		Rastrigin		Rosenbrock		Sphere	
Algorithm	GLSPSO	*R*-PSO_CLUS_	GLSPSO	*R*-PSO_CLUS_	GLSPSO	*R*-PSO_CLUS_	GLSPSO	*R*-PSO_CLUS_	GLSPSO	*R*-PSO_CLUS_

Best fitness	**3.5148**	20.9666	0.3195	**0.0022**	792.004	**293.5795**	**1378.0**	1867.2669	23.0614	**0.1970**
Mean fitness	**3.6709**	21.0691	0.4242	**0.0230**	881.0822	**688.0048**	**1602.0**	24909.8486	27.2534	**4.7232**
Worst fitness	**3.7664**	21.1306	0.4992	**0.0923**	934.9773	**848.9927**	**1763.0**	95519.4585	29.1615	**16.1174**
Std. Dev.	0.0551	**0.0316**	0.0303	**0.0255**	**35.2341**	103.1854	**90.2874**	21083.5791	**1.2253**	4.2498

**Table 7:** Comparison between GLSPSO and *L*-PSO_CLUS_ for problems with dimension of 10.

Problem	Ackley		Griewank		Rastrigin		Rosenbrock		Sphere	
Algorithm	GLSPSO	*L*-PSO_CLUS_	GLSPSO	*L*-PSO_CLUS_	GLSPSO	*L*-PSO_CLUS_	GLSPSO	*L*-PSO_CLUS_	GLSPSO	*L*-PSO_CLUS_

Best fitness	0.0364	**0.0000**	4.2879*e* − 04	0.0000**e** + 00	8.8062	**0.0000**	2.6188	**0.0000**	4.7832*e* − 04	6.4151**e** − 76
Mean fitness	**0.3413**	18.2504	**0.0041**	0.0042	29.4936	**0.0000**	9.0025	**1.0516**	0.0142	**0.0000**
Worst fitness	**1.2653**	20.0771	**0.0419**	0.1008	50.4781	**0.0000**	18.9887	**2.8033**	0.0476	**0.0000**
Std. Dev.	**0.2762**	5.4640	**0.0061**	0.0186	10.4372	**0.0000**	**0.034**	0.6449	0.0123	**0.0000**

**Table 8:** Comparison between GLSPSO and *L*-PSO_CLUS_ for problems with dimension of 30.

Problem	Ackley		Griewank		Rastrigin		Rosenbrock		Sphere	
Algorithm	GLSPSO	*L*-PSO_CLUS_	GLSPSO	*L*-PSO_CLUS_	GLSPSO	*L*-PSO_CLUS_	GLSPSO	*L*-PSO_CLUS_	GLSPSO	*L*-PSO_CLUS_

Best fitness	**2.2784**	20.3184	0.0897	**0.0000**	109.5946	**0.1444**	175.8785	**0.0000**	1.9123	**0.0000**
Mean fitness	**2.8398**	20.4631	0.1257	**0.0000**	185.5221	**18.7372**	218.4976	**25.1359**	2.7449	**0.0000**
Worst fitness	**3.2952**	20.5734	0.2074	**0.0000**	229.6229	**38.8433**	259.2466	**77.4444**	3.9559	**0.0000**
Std. Dev.	0.2273	**0.0615**	0.0274	**0.0000**	24.9829	**8.4570**	21.8027	**13.2536**	0.4840	**0.0000**

**Table 9:** Comparison between GLSPSO and *L*-PSO_CLUS_ for problems with dimension of 100.

Problem	Ackley		Griewank		Rastrigin		Rosenbrock		Sphere	
Algorithm	GLSPSO	*L*-PSO_CLUS_	GLSPSO	*L*-PSO_CLUS_	GLSPSO	*L*-PSO_CLUS_	GLSPSO	*L*-PSO_CLUS_	GLSPSO	*L*-PSO_CLUS_

Best fitness	**3.5148**	20.2136	0.3195	**0.0000**	792.004	**212.0416**	1378.0	**93.7390**	23.0614	**0.0000**
Mean fitness	**3.6709**	21.0491	0.4242	**0.0000**	881.0822	**366.6521**	1602.0	**107.2300**	27.2534	**0.0000**
Worst fitness	**3.7664**	21.1152	0.4992	**0.0000**	934.9773	**504.2204**	1763.0	**428.1758**	29.1615	**0.0000**
Std. Dev.	**0.0551**	0.1254	0.0303	**0.0000**	**35.2341**	68.2009	90.2874	**56.9231**	1.2253	**0.0000**

**Table 10:** Comparison between PSOlis, *R*-PSO_CLUS_ and *L*-PSO_CLUS_.

Problem	Algorithm		
	PSOlis	*R*-PSO_CLUS_	*L*-PSO_CLUS_
Ackley	4.081*e* − 03	9.263**e** − 13	3.7135**e** − 15
Griewank	2.673*e* − 02	6.921**e** − 03	2.945**e** − 07
Rastrigin	2.005	1.948**e** − 09	8.893**e** − 06
Rosenbrock	3.987	5.180**e** − 01	2.338**e** − 01
Sphere	6.137*e* − 14	2.197**e** − 27	2.401**e** − 54

**Table 11:** Results for RIW-PSO and *R*-PSO_CLUS_ for the 7 nonscaled benchmark problems.

Problem	Booth		Easom		Michalewicz		Schaffer's f6		Salomon		Shubert		Trid-6	
Algorithm	RIW-PSO	*R*-PSO_CLUS_	RIW-PSO	*R*-PSO_CLUS_	RIW-PSO	*R*-PSO_CLUS_	RIW-PSO	*R*-PSO_CLUS_	RIW-PSO	*R*-PSO_CLUS_	RIW-PSO	*R*-PSO_CLUS_	RIW-PSO	*R*-PSO_CLUS_

Best fitness	0.0000*e* + 00	0.0000*e* + 00	−1.0000*e* + 00	−1.0000*e* + 00	−3.3453*e* + 00	−4.4371**e** + 00	0.0000*e* + 00	0.0000*e* + 00	9.9833*e* − 02	7.3608**e** − 29	−1.8673*e* + 02	−1.8673*e* + 02	−5.0000*e* + 00	−5.0000*e* + 00
Mean fitness	0.0000*e* + 00	0.0000*e* + 00	−1.0000*e* + 00	−1.0000*e* + 00	−2.6034*e* + 00	−4.1008**e** + 00	4.7052*e* − 03	1.1659**e** − 03	9.9833*e* − 02	7.0073**e** − 02	−1.8673*e* + 02	−1.8673*e* + 02	−5.0000*e* + 00	−5.0000*e* + 00
Std. Dev.	0.0000*e* + 00	0.0000*e* + 00	6.6613*e* − 16	6.6613*e* − 16	4.2719*e* − 01	1.7866**e** − 01	4.8183*e* − 03	3.1573**e** − 03	6.2063**e** − 18	4.4236*e* − 02	3.7706**e** − 14	4.0594*e* − 14	8.6628*e* − 14	7.0555**e** − 14
Av. iteration	39.12	**37.92**	55.0	**45.48**	—	—	133.83	**109.95**	—	**923.40**	**71.8**	107.16	114.40	**110.20**
SR (%)	100	100	100	100	0	0	48	**88**	0	**20**	100	100	100	100

**Table 12:** Results for RIW-PSO and *R*-PSO_CLUS_ for the 14 scaled benchmark problems with dimension of 10.

Problem	Ackley		Griewank		Dixon-Price		Levy		Noisy Quartic		Noncontinuous Rastrigin		Rastrigin	
Algorithm	RIW-PSO	*R*-PSO_CLUS_	RIW-PSO	*R*-PSO_CLUS_	RIW-PSO	*R*-PSO_CLUS_	RIW-PSO	*R*-PSO_CLUS_	RIW-PSO	*R*-PSO_CLUS_	RIW-PSO	*R*-PSO_CLUS_	RIW-PSO	*R*-PSO_CLUS_

Best fitness	3.9968*e* − 15	4.4409**e** − 16	7.3960*e* − 03	0.0000**e** + 00	2.4652*e* − 31	2.4652*e* − 31	1.4997*e* − 32	1.4997*e* − 32	5.9308*e* − 04	2.7756**e** − 05	6.0002*e* + 00	0.0000**e** + 00	3.9767*e* + 00	0.0000**e** + 00
Mean fitness	1.0316*e* − 01	2.8599**e** − 15	6.6238*e* − 02	2.8539**e** − 03	6.1333*e* − 01	6.1333*e* − 01	4.1630*e* − 01	1.4997**e** − 32	4.6455*e* − 03	1.1590**e** − 04	1.1640*e* + 01	7.1054**e** − 17	1.2208*e* + 01	0.0000**e** + 00
Std. Dev.	5.0537*e* − 01	1.6573**e** − 15	3.3218*e* − 02	1.3981**e** − 02	1.8086*e* − 01	1.8086*e* − 01	7.5307*e* − 01	0.0000**e** + 00	3.2781*e* − 03	5.9530**e** − 05	4.3533*e* + 00	3.4809**e** − 16	4.4684*e* + 00	0.0000**e** + 00
Av. iteration	287.88	**263.68**	—	**464.16**	295.50	**258.50**	127.31	**99.32**	—	—	40.49	**12.44**	49.44	**25.00**
SR (%)	96	**100**	0	**96**	8	8	52	**100**	0	0	92	**100**	100	100

Problem	Rosenbrock		Rotated Ellipsoid		Schwefel		Schwefel 2.22		Sphere		Step		Sum Squares	

Algorithm	RIW-PSO	*R*-PSO_CLUS_	RIW-PSO	*R*-PSO_CLUS_	RIW-PSO	*R*-PSO_CLUS_	RIW-PSO	*R*-PSO_CLUS_	RIW-PSO	*R*-PSO_CLUS_	RIW-PSO	*R*-PSO_CLUS_	RIW-PSO	*R*-PSO_CLUS_

Best fitness	4.6541**e** − 03	3.7243*e* − 01	2.5690*e* − 27	5.9210**e** − 29	−3.2818*e* + 03	−4.1898**e** + 03	1.8348*e* − 32	2.7555**e** − 34	7.3673*e* − 61	5.4496**e** − 61	0.0000*e* + 00	0.0000*e* + 00	8.1835**e** − 62	1.9089*e* − 61
Mean fitness	1.4459*e* + 00	7.1832**e** − 01	1.0457*e* − 21	3.5437**e** − 24	−2.6199*e* + 03	−4.1384**e** + 03	3.3382*e* − 28	1.2830**e** − 30	4.1760*e* − 53	9.2787**e** − 55	8.0000*e* − 01	0.0000**e** + 00	2.1909*e* − 53	9.3329**e** − 54
Std. Dev.	1.6362*e* + 00	2.3127**e** − 01	3.0984*e* − 21	1.1617**e** − 23	3.3350*e* + 02	9.7987**e** + 01	1.3406*e* − 27	4.6701**e** − 30	1.2467*e* − 52	3.6764 − 54	1.9183 + 00	0.0000**e** + 00	1.0052*e* − 52	3.3839**e** − 53
Av. iteration	89.28	**35.16**	426.60	**337.16**	—	**877.69**	268.84	**222.04**	180.12	**158.08**	88.72	**15.68**	143.52	**123.48**
SR (%)	100	100	100	100	0	**52**	100	100	100	100	72	**100**	100	100

**Table 13:** Results for RIW-PSO and *R*-PSO_CLUS_ for the 14 scaled benchmark problems with dimension of 20.

Problem	Ackley		Griewank		Dixon-Price		Levy		Noisy Quartic		Noncontinuous Rastrigin		Rastrigin	
Algorithm	RIW-PSO	*R*-PSO_CLUS_	RIW-PSO	*R*-PSO_CLUS_	RIW-PSO	*R*-PSO_CLUS_	RIW-PSO	*R*-PSO_CLUS_	RIW-PSO	*R*-PSO_CLUS_	RIW-PSO	*R*-PSO_CLUS_	RIW-PSO	*R*-PSO_CLUS_

Best fitness	7.5495*e* − 15	3.9968**e** − 15	0.0000*e* + 00	0.0000**e** + 00	6.6667*e* − 01	6.6667*e* − 01	1.4997*e* − 32	1.4997*e* − 32	3.9758*e* − 03	8.2784**e** − 05	1.2000*e* + 01	1.6698**e** − 03	1.4000*e* + 01	9.8524**e** − 03
Mean fitness	2.6017*e* − 01	3.9968**e** − 15	2.7935*e* − 02	0.0000**e** + 00	6.6667*e* − 01	6.6667*e* − 01	7.8421*e* − 01	1.4997**e** − 32	1.0286*e* − 02	2.7771**e** − 04	3.0521*e* + 01	4.4448**e** + 00	2.8521*e* + 01	4.3599**e** + 00
Std. Dev.	6.0386*e* − 01	0.0000**e** + 00	1.8216*e* − 02	0.0000**e** + 00	2.4222**e** − 16	4.1352*e* − 08	8.0230*e* − 01	0.0000**e** + 00	5.0796*e* − 03	1.3566**e** − 04	1.0922*e* + 01	2.5668**e** + 00	9.5087*e* + 00	1.9684**e** + 00
Av. iteration	580.67	**488.52**	402.00	**382.80**	—	—	248.00	**200.04**	—	—	51.5	**86.72**	56.2	89.08
SR (%)	84	**100**	4	**100**	0	0	16	**100**	0	0	16	**100**	20	**100**

Problem	Rosenbrock		Rotated Ellipsoid		Schwefel		Schwefel 2.22		Sphere		Step		Sum Squares	

Algorithm	RIW-PSO	*R*-PSO_CLUS_	RIW-PSO	*R*-PSO_CLUS_	RIW-PSO	*R*-PSO_CLUS_	RIW-PSO	*R*-PSO_CLUS_	RIW-PSO	*R*-PSO_CLUS_	RIW-PSO	*R*-PSO_CLUS_	RIW-PSO	*R*-PSO_CLUS_

Best fitness	8.2827**e** − 01	7.4672*e* + 00	8.7694*e* − 04	7.9922**e** − 09	−5.9318*e* + 03	−8.1903**e** + 03	2.4775*e* − 22	3.5223**e** − 26	3.1840*e* − 43	2.9531**e** − 44	0.0000*e* + 00	0.0000**e** + 00	4.8722*e* − 45	2.1277**e** − 46
Mean fitness	9.1608**e** + 00	1.0488*e* + 01	7.2146**e** − 06	1.9591*e* − 05	−4.5638*e* + 03	−7.0464**e** + 03	8.5083*e* − 17	4.9420**e** − 23	3.9759*e* − 37	8.4151**e** − 38	1.5600*e* + 00	0.0000**e** + 00	8.3393*e* − 39	1.4859**e** − 39
Std. Dev.	3.5710*e* + 00	8.1620**e** − 01	1.9200**e** − 05	7.2466*e* − 05	6.0867*e* + 02	8.4004**e** + 02	3.9686*e* − 16	1.2554**e** − 22	1.2218*e* − 36	3.0436**e** − 37	1.6020*e* + 00	0.0000**e** + 00	1.4280*e* − 38	4.9918**e** − 39
Av. iteration	263.40	**133.72**	1763.10	**1636.74**	—	—	575.92	**418.88**	354.00	**309.04**	646.57	**19.36**	310.72	**268.12**
SR (%)	100	100	84	**92**	0	0	100	100	100	100	28	**100**	100	100

**Table 14:** Results for RIW-PSO and *R*-PSO_CLUS_ for the 14 scaled benchmark problems with dimension of 30.

Problem	Ackley		Griewank		Dixon-Price		Levy		Noisy Quartic		Noncontinuous Rastrigin		Rastrigin	
Algorithm	RIW-PSO	*R*-PSO_CLUS_	RIW-PSO	*R*-PSO_CLUS_	RIW-PSO	*R*-PSO_CLUS_	RIW-PSO	*R*-PSO_CLUS_	RIW-PSO	*R*-PSO_CLUS_	RIW-PSO	*R*-PSO_CLUS_	RIW-PSO	*R*-PSO_CLUS_

Best fitness	1.4655*e* − 14	3.9968**e** − 15	0.0000*e* + 00	0.0000**e** + 00	6.6667*e* − 01	6.6667*e* − 01	2.6858*e* − 01	1.4997**e** − 32	3.3454*e* − 03	1.0873**e** − 04	1.9001*e* + 01	5.7746**e** − 02	1.7895*e* + 01	0.0000**e** + 00
Mean fitness	5.3345*e* − 01	4.1389**e** − 15	1.1200*e* − 02	0.0000**e** + 00	6.6667*e* − 01	6.6716*e* − 01	1.8286*e* + 00	1.4997**e** − 32	1.1252*e* − 02	3.1438**e** − 04	4.3331*e* + 01	9.0806**e** + 00	3.2489*e* + 01	6.5810**e** + 00
Std. Dev.	8.1543*e* − 01	6.9619**e** − 16	1.4757*e* − 02	0.0000**e** + 00	3.2634**e** − 16	2.4146*e* − 03	1.1781*e* + 00	0.0000**e** + 00	4.3926*e* − 03	1.1754**e** − 04	1.8889*e* + 01	5.1000**e** + 00	1.0662*e* + 01	5.7209**e** + 00
Av. iteration	959.88	**776.20**	628.00	**525.48**	—	—	—	**348.76**	—	—	107	**377.72**	141.00	**379.35**
SR (%)	64	**100**	36	**100**	0	0	0	**100**	0	0	4	**100**	4	**92**

Problem	Rosenbrock		Rotated Ellipsoid		Schwefel		Schwefel 2.22		Sphere		Step		Sum Squares	

Algorithm	RIW-PSO	*R*-PSO_CLUS_	RIW-PSO	*R*-PSO_CLUS_	RIW-PSO	*R*-PSO_CLUS_	RIW-PSO	*R*-PSO_CLUS_	RIW-PSO	*R*-PSO_CLUS_	RIW-PSO	*R*-PSO_CLUS_	RIW-PSO	*R*-PSO_CLUS_

Best fitness	4.0555**e** − 02	1.8942*e* + 01	3.2599*e* − 04	1.7148**e** − 04	−7.9654*e* + 03	−1.0692**e** + 04	2.7819*e* − 17	1.0011**e** − 24	5.7947*e* − 39	5.0490**e** − 42	0.0000*e* + 00	0.0000*e* + 00	1.7310*e* − 36	1.2387**e** − 44
Mean fitness	2.3794*e* + 01	1.9930**e** + 01	5.5035**e** − 03	1.8327*e* − 02	−6.7376*e* + 03	−9.1033**e** + 03	4.1597*e* − 13	6.4368**e** − 23	4.1277*e* − 33	3.6673**e** − 36	3.7600*e* + 00	0.0000**e** + 00	3.9240*e* − 33	5.9219**e** − 39
Std. Dev.	1.9052*e* + 01	5.2276**e** − 01	8.1952**e** − 03	4.4826*e* − 02	8.7747*e* + 02	8.6528**e** + 02	1.5547*e* − 12	7.6462**e** − 23	1.1473*e* − 32	8.2055**e** − 36	2.3200*e* + 00	0.0000**e** + 00	1.3909*e* − 32	1.2917**e** − 38
Av. iteration	1523.36	2813.14	—	—	—	—	1139.64	**658.68**	595.52	**505.80**	2881.00	**21.48**	526.28	**436.08**
SR (%)	44	**56**	0	0	0	0	100	100	100	100	4	**100**	100	100

**Table 15:** Results for LDIW-PSO and *L*-PSO_CLUS_ for the 7 nonscaled benchmark problems.

Problem	Booth		Easom		Michalewicz		Schaffer's f6		Salomon		Shubert		Trid-6	
Algorithm	LDIW-PSO	*L*-PSO_CLUS_	LDIW-PSO	*L*-PSO_CLUS_	LDIW-PSO	*L*-PSO_CLUS_	LDIW-PSO	*L*-PSO_CLUS_	LDIW-PSO	*L*-PSO_CLUS_	LDIW-PSO	*L*-PSO_CLUS_	LDIW-PSO	*L*-PSO_CLUS_

Best fitness	0.0000*e* + 00	0.0000*e* + 00	−1.0000*e* + 00	−1.0000*e* + 00	−3.4792*e* + 00	−4.3762**e** + 00	0.0000*e* + 00	0.0000*e* + 00	9.9833*e* − 02	3.3189**e** − 65	−1.8673*e* + 02	−1.8673*e* + 02	−5.0000*e* + 01	−5.0000*e* + 01
Mean fitness	0.0000*e* + 00	0.0000*e* + 00	−1.0000*e* + 00	−1.0000*e* + 00	−2.6736*e* + 00	−4.1056**e** + 00	1.5545*e* − 03	1.1659**e** − 03	9.9833*e* − 02	8.7853**e** − 02	−1.8673*e* + 02	−1.8673*e* + 02	−50000*e* + 01	−50000*e* + 01
Std. Dev.	0.0000*e* + 00	0.0000*e* + 00	6.6613*e* − 16	6.6613*e* − 16	3.6921*e* − 01	1.6196**e** − 01	3.5619*e* − 03	3.1573**e** − 03	7.3434**e** − 18	3.2442*e* − 02	3.9382**e** − 14	7.5559*e* − 05	7.8572**e** − 14	9.3154*e* − 14
Av. iteration	**79.48**	81.16	107.16	**73.12**	—	—	279.90	**174.14**	—	**742**	**175.52**	208.20	287.48	**281.72**
SR (%)	100	100	100	100	0	0	84	**88**	0	**12**	**100**	96	100	100

**Table 16:** Results for LDIW-PSO and *L*-PSO_CLUS_ for the 14 scaled benchmark problems with dimension of 10.

Problem	Ackley		Griewank		Dixon-Price		Levy		Noisy Quartic		Noncontinuous Rastrigin		Rastrigin	
Algorithm	LDIW-PSO	*L*-PSO_CLUS_	LDIW-PSO	*L*-PSO_CLUS_	LDIW-PSO	*L*-PSO_CLUS_	LDIW-PSO	*L*-PSO_CLUS_	LDIW-PSO	*L*-PSO_CLUS_	LDIW-PSO	*L*-PSO_CLUS_	LDIW-PSO	*L*-PSO_CLUS_

Best fitness	3.9968*e* − 15	4.4409**e** − 16	2.9510*e* − 02	0.0000**e** + 00	6.6667*e* − 01	6.6667*e* − 01	1.4997*e* − 31	1.4997**e** − 32	3.4641 − 04	1.1331**e** − 05	4.0001*e* + 00	0.0000**e** + 00	2.9825*e* + 00	0.0000**e** + 00
Mean fitness	4.9916*e* − 15	2.4335**e** − 15	8.4720*e* − 02	1.4639**e** − 06	6.6667*e* − 01	6.6667*e* − 01	1.9892*e* − 01	1.4997**e** − 32	2.9606*e* − 03	9.7778**e** − 05	1.2200*e* + 01	4.0007**e** − 02	9.9019*e* + 00	0.0000**e** + 00
Std. Dev.	1.5952**e** − 15	1.7635*e* − 15	2.7646*e* − 02	7.1705**e** − 06	1.5401*e* − 10	1.2278**e** − 12	4.4094*e* − 01	0.0000**e** + 00	2.0277*e* − 03	6.3873**e** − 05	6.2484*e* + 00	1.9600**e** − 01	5.2190*e* + 00	0.0000**e** + 00
Av. iteration	500.92	**486.84**	—	**703.83**	—	—	309.58	**281.36**	—	—	40.86	**12.88**	45.13	**26.24**
SR (%)	100	100	0	**96**	0	0	76	**100**	0	0	88	**100**	96	**100**

Problem	Rosenbrock		Rotated Ellipsoid		Schwefel		Schwefel 2.22		Sphere		Step		Sum Squares	

Algorithm	LDIW-PSO	*L*-PSO_CLUS_	LDIW-PSO	*L*-PSO_CLUS_	LDIW-PSO	*L*-PSO_CLUS_	LDIW-PSO	*L*-PSO_CLUS_	LDIW-PSO	*L*-PSO_CLUS_	LDIW-PSO	*L*-PSO_CLUS_	LDIW-PSO	*L*-PSO_CLUS_

Best fitness	2.7701**e** − 03	1.5581*e* − 01	2.1928*e* − 44	1.2232**e** − 52	−3.2028*e* + 03	−4.1898**e** + 03	3.3962**e** − 48	2.3988*e* − 46	9.8201*e* − 110	3.2307**e** − 112	0.0000*e* + 00	0.0000*e* + 00	2.3361*e* − 110	1.6446**e** − 112
Mean fitness	9.3256*e* − 01	7.2655**e** − 01	5.8802*e* − 32	4.6946**e** − 47	−2.5079*e* + 03	−4.1317**e** + 03	8.0458*e* − 23	5.4599**e** − 29	3.1249*e* − 101	1.7383**e** − 106	1.6000*e* − 01	0.0000**e** + 00	1.6238**e** − 105	6.7305*e* − 105
Std. Dev.	1.3984*e* + 00	5.1516**e** − 01	2.8672*e* − 31	1.6736**e** − 46	4.0568*e* + 02	1.2489**e** + 02	3.9336*e* − 22	2.6683**e** − 28	1.2642*e* − 100	3.3735**e** − 106	7.8384*e* − 01	0.0000**e** + 00	5.1760**e** − 105	3.1425*e* − 104
Av. iteration	155.20	**69.64**	566.44	**559.20**	—	**798.45**	478.12	**450.76**	385.52	**374.28**	73.79	**15.76**	340.56	**327.24**
SR (%)	100	100	100	100	0	**44**	100	100	100	100	96	**100**	100	100

**Table 17:** Results for LDIW-PSO and *L*-PSO_CLUS_ for the 14 scaled benchmark problems with dimension of 20.

Problem	Ackley		Griewank		Dixon-Price		Levy		Noisy Quartic		Noncontinuous Rastrigin		Rastrigin	
Algorithm	LDIW-PSO	*L*-PSO_CLUS_	LDIW-PSO	*L*-PSO_CLUS_	LDIW-PSO	*L*-PSO_CLUS_	LDIW-PSO	*L*-PSO_CLUS_	LDIW-PSO	*L*-PSO_CLUS_	LDIW-PSO	*L*-PSO_CLUS_	LDIW-PSO	*L*-PSO_CLUS_

Best fitness	1.4655*e* − 14	3.9968**e** − 15	7.3960*e* − 03	0.0000**e** + 00	6.6667*e* − 01	6.6667*e* − 01	1.4997*e* − 32	1.4997*e* − 32	8.8724*e* − 04	8.2862**e** − 05	9.0003*e* + 00	5.4958**e** − 02	7.8533*e* + 00	0.0000**e** + 00
Mean fitness	2.7869*e* − 13	3.9968**e** − 15	3.5766*e* − 02	1.1969**e** − 05	6.6667*e* − 01	6.6667*e* − 01	9.4811*e* − 01	1.4997**e** − 32	6.2935*e* − 03	1.8753**e** − 04	2.9441*e* + 01	5.0733**e** + 00	2.2627*e* + 01	1.5954**e** + 00
Std. Dev.	1.1016*e* − 12	0.0000**e** + 00	2.3388*e* − 02	3.7730**e** − 05	9.4022**e** − 16	2.6746*e* − 08	1.0290*e* + 00	0.0000**e** + 00	2.8130*e* − 03	9.1506**e** − 05	1.1371*e* + 01	2.4613**e** + 00	7.0594*e* + 00	2.9944**e** + 00
Av. iteration	785.08	**773.48**	—	**813.50**	—	—	545.13	**518.48**	—	—	93	**88.40**	256.17	**144.32**
SR (%)	100	100	0	**88**	0	0	32	**100**	0	0	16	**100**	48	**100**

Problem	Rosenbrock		Rotated Ellipsoid		Schwefel		Schwefel 2.22		Sphere		Step		Sum Squares	

Algorithm	LDIW-PSO	*L*-PSO_CLUS_	LDIW-PSO	*L*-PSO_CLUS_	LDIW-PSO	*L*-PSO_CLUS_	LDIW-PSO	*L*-PSO_CLUS_	LDIW-PSO	*L*-PSO_CLUS_	LDIW-PSO	*L*-PSO_CLUS_	LDIW-PSO	*L*-PSO_CLUS_

Best fitness	8.5273**e** − 01	7.8279*e* + 00	5.7451*e* − 10	3.1410**e** − 20	−5.5568*e* + 03	−79200**e** + 03	2.1114*e* − 17	4.5537**e** − 28	3.4892*e* − 59	4.4204**e** − 68	0.0000*e* + 00	0.0000*e* + 00	2.7411*e* − 60	3.2424**e** − 71
Mean fitness	8.8496**e** + 00	9.1318*e* + 00	6.1269*e* − 05	1.7093**e** − 15	−4.5047*e* + 03	−68180**e** + 03	3.5766*e* − 09	1.6526**e** − 23	1.0393*e* − 45	1.6274**e** − 58	8.0000*e* − 02	0.0000**e** + 00	1.4838*e* − 48	1.5378**e** − 59
Std. Dev.	3.4651*e* + 00	9.4307**e** − 01	1.7066*e* − 04	4.8692**e** − 15	5.0603 + 02	5.1886*e* + 02	8.6693*e* − 09	3.1511**e** − 23	3.1193*e* − 45	5.5196**e** − 58	2.7129*e* − 01	0.0000**e** + 00	5.6876*e* − 48	7.4256**e** − 59
Av. iteration	594.96	419.12	1495.45	**1254.52**	—	—	820.56	**722.76**	639.88	**636.08**	147.52	**19.88**	595.80	**593.52**
SR (%)	100	100	88	**100**	0	0	100	100	100	100	92	**100**	100	100

**Table 18:** Results for LDIW-PSO and *L*-PSO_CLUS_ for the 14 scaled benchmark problems with dimension of 30.

Problem	Ackley		Griewank		Dixon-Price		Levy		Noisy Quartic		Noncontinuous Rastrigin		Rastrigin	
Algorithm	LDIW-PSO	*L*-PSO_CLUS_	LDIW-PSO	*L*-PSO_CLUS_	LDIW-PSO	*L*-PSO_CLUS_	LDIW-PSO	*L*-PSO_CLUS_	LDIW-PSO	*L*-PSO_CLUS_	LDIW-PSO	*L*-PSO_CLUS_	LDIW-PSO	*L*-PSO_CLUS_

Best fitness	3.6993*e* − 13	3.9968**e** − 15	2.2204*e* − 16	0.0000**e** + 00	6.6667*e* − 01	6.6667*e* − 01	3.4554 − 28	1.4997**e** − 32	3.2281*e* − 03	8.0745**e** − 05	2.0001*e* + 01	2.2356**e** + 00	1.8889*e* + 01	0.0000**e** + 00
Mean fitness	5.6071*e* − 11	3.9968**e** − 15	1.2289*e* − 02	0.0000**e** + 00	6.6667*e* − 01	6.6667*e* − 01	2.0125*e* + 00	1.4997**e** − 32	7.2161*e* − 03	2.3200**e** − 04	4.1361*e* + 01	1.2883**e** + 01	3.3921*e* + 01	8.3217**e** + 00
Std. Dev.	2.1743*e* − 10	0.0000**e** + 00	1.5738*e* − 02	0.0000**e** + 00	3.8233*e* − 12	3.1157**e** − 14	1.6464*e* + 00	0.0000**e** + 00	3.4677*e* − 03	1.0514**e** − 04	1.3494*e* + 01	5.1402**e** + 00	9.7583 + 00	7.6697**e** + 00
Av. iteration	1245.68	**1194.40**	1050.23	1060.44	—	—	928.00	**865.44**	—	—	—	**658.48**	518.00	718.21
SR (%)	100	100	52	**100**	0	0	4	**100**	0	0	0	**92**	4	**96**

Problem	Rosenbrock		Rotated Ellipsoid		Schwefel		Schwefel 2.22		Sphere		Step		Sum Squares	

Algorithm	LDIW-PSO	*L*-PSO_CLUS_	LDIW-PSO	*L*-PSO_CLUS_	LDIW-PSO	*L*-PSO_CLUS_	LDIW-PSO	*L*-PSO_CLUS_	LDIW-PSO	*L*-PSO_CLUS_	LDIW-PSO	*L*-PSO_CLUS_	LDIW-PSO	*L*-PSO_CLUS_

Best fitness	1.4208**e** − 01	1.4715*e* + 01	1.4959*e* − 04	3.8064**e** − 11	−7.7298*e* + 03	−1.0232**e** + 04	6.6416*e* − 10	1.2902**e** − 24	9.3155*e* − 41	1.4592**e** − 57	0.0000*e* + 00	0.0000*e* + 00	1.0873*e* − 41	1.2031**e** − 57
Mean fitness	1.8350*e* + 01	1.7789**e** + 01	4.5745*e* − 02	1.8073**e** − 06	−6.5026*e* + 03	−8.6435**e** + 03	1.0569*e* − 04	2.4455**e** − 22	1.2325*e* − 30	6.2108**e** − 47	7.2000*e* − 01	0.0000**e** + 00	1.6409*e* − 28	2.0496**e** − 48
Std. Dev.	1.1240*e* + 01	1.8867**e** + 00	1.3691*e* − 01	4.7189**e** − 06	7.1966**e** + 02	1.0203*e* + 03	4.0792*e* − 04	2.8267**e** − 22	3.7363*e* − 30	3.0419**e** − 46	8.7270*e* − 01	0.0000**e** + 00	5.9071*e* − 28	9.3904**e** − 48
Av. iteration	**1920.22**	2078.70	—	**2524.35**	—	—	1576.75	**1120.20**	1033.04	**1028.12**	408.92	**22.52**	968.72	**952.28**
SR (%)	92	92	0	**92**	0	0	80	**100**	100	100	52	**100**	100	100

**Table 19:** Wilcoxon signed rank test on mean fitness obtained by RIW-PSO and *R*-PSO_CLUS _for the test problems.

Measurement	Scaled problems			Nonscaled problems
	Dim = 10	Dim = 20	Dim = 30	
*R*-PSO_CLUS _< RIW-PSO	13	11	12	3
*R*-PSO_CLUS _> RIW-PSO	0	2	2	0
*R*-PSO_CLUS_ = RIW-PSO	1	1	0	4
*z*	−3.190	−2.274	−2.606	−1.604
*P* value	0.001	0.023	0.009	0.190
*r*	0.600	0.430	0.490	—
Median				
RIW-PSO	0.847	0.144	0.272	−1.000
*R*-PSO_CLUS_	0.000	0.000	0.000	−1.000

**Table 20:** Wilcoxon signed rank test on mean fitness obtained by LDIW-PSO and *L*-PSO_CLUS _for the test problems.

Measurement	Scaled problems			Nonscaled
	Dim = 10	Dim = 20	Dim = 30	
*L*-PSO_CLUS_ < LDIW-PSO	12	12	13	3
*L*-PSO_CLUS_ > LDIW-PSO	1	1	0	0
*L*-PSO_CLUS_ = LDIW-PSO	1	1	1	4
*z*	−2.988	−2.552	−3.181	−1.604
*P* value	0.003	0.011	0.001	0.190
*r*	0.565	0.482	0.601	—
Median				
LDIW-PSO	0.044	0.021	0.029	−1.000
*L*-PSO_CLUS _	0.000	0.000	0.000	−1.000
